# High cyclin B1 expression is associated with poor survival in breast cancer

**DOI:** 10.1038/sj.bjc.6604874

**Published:** 2009-03-17

**Authors:** K Aaltonen, R-M Amini, P Heikkilä, K Aittomäki, A Tamminen, H Nevanlinna, C Blomqvist

**Affiliations:** 1 Department of Oncology, Helsinki University Central Hospital, P.O. Box 180, Helsinki FI-00029 HUS, Finland; 2 Department of Obstetrics and Gynaecology, Helsinki University Central Hospital, P.O. Box 140, Helsinki FI-00029 HUS, Finland; 3 Department of Genetics and Pathology, Uppsala University Hospital, Uppsala SE-75185, Sweden; 4 Department of Pathology, Helsinki University Central Hospital, P.O. Box 400, Helsinki FI-00029 HUS, Finland; 5 Department of Clinical Genetics, Helsinki University Central Hospital, P.O. Box 140, Helsinki FI-00029 HUS, Finland; 6 Department of Oncology, Radiology and Clinical Immunology, Uppsala University Hospital, Uppsala SE-75185, Sweden

**Keywords:** breast cancer, cyclin B1, survival, prognosis

## Abstract

Cyclin B1 regulates the G_2_-M transition of the cell cycle. Cyclin B1 expression is higher in premalignant and malignant than normal breast lesions. Correlation of cyclin B1 expression with other histopathological variables and prognostic role in breast cancer are not fully understood. Traditionally used prognostic criteria identify large subset of patients to receive adjuvant chemotherapy and to be exposed to adverse effects. A reliable and simple method helping prognostic evaluation in breast cancer is needed. We analysed cyclin B1 expression on 1348 invasive breast cancers and studied correlations with other histopathological variables and survival. High cyclin B1 correlated with high tumour grade, large tumour size and positive nodal status, oestrogen and progesterone receptor negativity, positive HER2 and p53 status, young age at diagnosis, and high cyclin E, cyclin A and Ki67 expression. Among patients not given adjuvant chemotherapy high cyclin B1 was a strong predictor of shorter overall and metastasis-free survival (RR 3.74, *P*<0.0005 and RR 3.51, *P*<0.0005, respectively), and remained as an independent prognostic factor also in multivariate analysis (RR 1.80, *P*=0.04 and RR 2.31, *P*=0.02, respectively). This study suggests high cyclin B1 associates with aggressive phenotype and is an independent prognostic factor in breast cancer.

Breast cancer is a heterogeneous disease with varying biological profile and aggressiveness. Adjuvant chemotherapy improves breast cancer survival but can cause side effects that may potentially have negative long-term effects on quality of life of women surviving breast cancer. Therefore additional markers are needed for better selection of the patients benefiting from adjuvant treatment and to avoid overtreatment. Traditional prognostic factors, such as tumour size, lymph node status and tumour grade are used to identify patients with high risk for recurrence who potentially benefit from adjuvant chemotherapy (Bast *et al*, 2001). Negative hormone receptor and positive HER2 status also predict poor outcome. A new entity, the so-called triple negative (oestrogen receptor, progesterone receptor and HER2 negative) breast cancer has a highly aggressive clinical course with shorter recurrence-free and overall survival (Dent *et al*, 2007). High proliferation rate has also been shown to associate with poor breast cancer survival (Dettmar *et al*, 1997; Bryant *et al*, 1998; Ahlin *et al*, 2007). Newer methods utilising complementary DNA (cDNA) have been developed aiming at more specific prognostic evaluation than by immunohistochemical methods (van de Vijver *et al*, 2002; van’t Veer *et al*, 2002; Paik *et al*, 2004). These methods are laborious and expensive, and are not as easily adapted for routine use as are immunohistochemical methods.

Carefully regulated expression of cyclins and cyclin-dependent kinases controls the cell cycle (Sherr, 1996). The cyclin B1/CDK1 complex controls the G_2_-M phase transition, and is needed for initiation of mitosis (Pines and Hunter, 1990). Deregulation of cyclin B1 causes uncontrolled cell growth and may promote malignant transformation. p53 regulates G_2_-M transition through cyclin B1 expression level (Innocente *et al*, 1999). In cancer cells, cyclin B1 expression has also been detected in G_1_ phase (Shen *et al*, 2004). This continuous, unscheduled expression may lead to substrate phosphorylation regardless of the cell-cycle phase and thus cause uncontrolled cell-cycle progression and be one of the mechanisms in genetic instability and carcinogenesis.

Cyclin B1 overexpression is common in several cancers (Murakami *et al*, 1999; Soria *et al*, 2000; Nozoe *et al*, 2002; Takeno *et al*, 2002; Yoshida *et al*, 2004; Ikuerowo *et al*, 2006). It has been shown to associate with high-grade tumours and advanced stage of disease, as well as poor prognosis, in several cancers including oesophageal squamous cell (Murakami *et al*, 1999; Nozoe *et al*, 2002; Takeno *et al*, 2002), non-small cell lung (Soria *et al*, 2000; Yoshida *et al*, 2004) and renal cell cancer (Ikuerowo *et al*, 2006).

Cyclin B1 expression level increases in the transition from benign through premalignant to advanced malignant breast lesions (Kawamoto *et al*, 1997). The first reported study of cyclin B1 expression in breast cancer comprised only 73 cancers (Winters *et al*, 2001). Both nuclear and cytoplasmic expressions were independent predictors of poor relapse-free and overall survival. Cyclin B1 expression was not associated with tumour size, nodal status, grade, oestrogen receptor (ER) status or p53 immunohistochemical expression. In another study with 332 T1-2 N-negative breast cancers (Kühling *et al*, 2003; Rudolph *et al*, 2003), high cyclin B1 expression associated with high grade, high Ki67, cyclin A and E expression, and ER and progesterone receptor (PR) negativity and predicted relapse-free and overall survival in univariate analysis but was not an independent prognostic factor in multivariate analysis including Ki67 as a covariate (Rudolph *et al*, 2003). Among the 273 tumours treated with surgery and postoperative radiation only, cyclin B1 was an independent predictor of poor overall survival among premenopausal but not postmenopausal or all patients (Kühling *et al*, 2003). A further study with 56 invasive stage I–II cancers did not show any association between cyclin B1 expression and prognosis (Peters *et al*, 2004). A recent study with 109 breast cancers suggested that nuclear cyclin B1 expression was an independent prognostic factor (Suzuki *et al*, 2007).

The studies of cyclin B1 expression in breast cancer strongly suggest a prognostic role but these studies have been rather small. In this study we investigated cyclin B1 expression, its correlation with other histopathological features and survival in an extensive series of 1348 breast cancers (779 cancers in survival analysis).

## Materials and methods

### Patients

The study consists of 1348 invasive breast cancers. Of these, 884 are unselected patients treated at the Department of Oncology, Helsinki University Central Hospital between 1997–1998 and 2000 (79% of all consecutive, newly diagnosed breast cancer cases during the collection periods; Syrjäkoski *et al*, 2000; Kilpivaara *et al*, 2005). The rest of the patients are familial breast cancer patients identified by systematic screening at the Department of Oncology, Helsinki University Central Hospital or ascertained through genetic counselling at the Department of Clinical Genetics (Eerola *et al*, 2000). Of all patients, 439 are sporadic, 456 have strong family history (at least three first or second degree relatives with breast or ovarian cancer, including the proband), 342 have family history of two affected first degree relatives (including the proband) and 53 patients are BRCA1 and 58 BRCA2 mutation carriers.

Information on tumour histology, grade, size, nodal status, distant metastases, ER and PR status were obtained from pathology reports (Eerola *et al*, 2005). An expert breast cancer pathologist re-reviewed all tumours for histology and grade. Grading was performed according to Scarff–Bloom–Richardson modified by Elston and Ellis. Patient characteristics are shown in Table 1. Tissue microarray (TMA) construction has been described earlier (Eerola *et al*, 2005). HER2 expression was analysed by immunohistochemical staining and gene amplification by chromogenic *in situ* hybridisation on TMAs (Tanner *et al*, 2000; Lassus *et al*, 2004), and p53 (Tommiska *et al*, 2005), cyclin A (Aaltonen *et al*, 2006) and Ki67 (Ahlin *et al*, 2007) protein expression by immunohistochemical staining as previously described.

### Immunohistochemistry

After deparaffinisation in xylene and hydration in graded alcohols, cyclin B1 immunostaining was done in automated immunostainer (Ventana Medical Systems Inc., Tucson, AZ, USA) using a diaminobenzidine kit and amplification kit (Ventana) to ensure standardised performance. Cyclin B1 antibody (Novocastra, Newcastle-upon-Tyne, UK) was diluted 1 : 40 and antigen retrieval was done using the iView kit (Ventana). Only unequivocal positive nuclear or cytoplasmic staining was accepted as a positive reaction, and cyclin B1 result was the percentage of tumour cells displaying cytoplasmic or nuclear immunoreactivity. Normal breast tissue specimen was used as negative and human palatine tonsil tissue specimen as positive control for cyclin B1. Minimum of 500 tumour cells on each tumour was calculated ( × 40 objective). One investigator analysed the TMAs. All scoring was done under the supervision of an expert breast cancer pathologist. Result was obtained from 1100 tumours (81.4%). Cyclin B1 median value was 5.0%, range 0–71.5%, standard deviation 6.39 and standard error 0.19. Cyclin B1 expression followed the normal distribution.

### The follow-up data

Information on adjuvant treatment and distant metastases during the follow-up was collected from the patient records. The information on death due to breast cancer or other reason was obtained from the Finnish Cancer Registry. Survival was analysed as metastasis-free survival (MFS): the time from the date of primary surgery to the date of radiological confirmed distant metastases, and as overall survival (OS): the time from the date of primary surgery to the date of death due to breast cancer. A total of 797 patients were accepted for survival analysis: including the unselected series and familial patients ascertained to the familial breast cancer study at the diagnosis or within 6 months after diagnosis. Of these 797 patients, 796 (99.9%) underwent surgery, 691 (87%) received adjuvant radiotherapy, 323 (41%) adjuvant chemotherapy and 359 (45%) adjuvant endocrine treatment. Of the patients that were given adjuvant chemotherapy, 163 (50%) were treated with CMF (cyclophosphamide-methotrexate-5-fluorouracil), 102 (32%) with CEF (cyclophosphamide-epirubicin-5-fluorouracil) and 58 (18%) with some other chemotherapy regimen. Chemotherapy, endocrine treatment and radiation were given postoperative. Treatment decisions were made according to standard guidelines at that time. The median follow-up time was 93 months (2–516 months). Of all the patients in the survival analysis, 127 (16%) relapsed with distant metastases during the follow-up time, of whom 91 (11%) died from breast cancer.

### Statistical analyses

Statistical analyses were assessed with SPSS for Windows v12.0.1 (SPSS Inc., Chicago, IL, USA) and SISA (http://home.clara.net/sisa/). Correlation of cyclin B1 expression (as a continuous variable) and other histopathological features was assessed with Mann–Whitney *U*-test (dichotomised variables) or Spearman's *σ*-correlation test (continuous variables). The frequencies of cyclin B1 positive tumours among patient groups with different familial background were compared with *χ*^2^-test. A study investigating the optimal cut-off values of cyclin A and Ki67 for prognostic evaluation suggested that cut-off value around the 7th decile gives best separation between slowly and rapidly proliferating tumours (Ahlin *et al*, 2007). The relative risk (RR) for MFS and OS with 95% confidence interval (CI) using the Cox proportional hazard model was calculated for cyclin B1 dichotomised at 7th decile (cut-off value 5.6%). Kaplan–Meier curves were constructed for survival comparing the subsets of cases using a log-rank test. All *P*-values are two-sided and significance level is 0.05.

### Ethics

This study was performed with informed consent from the patients as well as permission from the Ethics Committee of the Helsinki University Central Hospital and from the Ministry of Social Affairs and Health in Finland.

## Results

High cyclin B1 expression was associated with large tumour size, positive nodal status, advanced clinical stage, high grade, ER and PR negativity, positive p53, HER2, and Ki67 status, high cyclin A and cyclin E expression, younger age at disease onset, and premenopausal status. Furthermore high cyclin B1 expression was significantly more common among triple-negative tumours. Cyclin B1 and D1 expressions did not correlate. Table 2 shows the correlations between cyclin B1 and other tumour features. Ductal and medullary histology were significantly more common among tumours with high than low cyclin B1 expression (*P*<0.0005 and *P*=0.0008, respectively), and lobular histology among tumours with low cyclin B1 expression (*P*<0.0005).

Tumours with the highest cyclin B1 expression (>10%) were more frequent among BRCA1 than sporadic (OR 2.8, 95% CI 1.4–5.6, *P*=0.003) or familial BRCA1/2 mutation negative (OR 4.8, 95% CI 2.3–9.9, *P*<0.0005) patients. Cyclin B1 expression among BRCA2-related tumours did not significantly differ from expression among tumours of sporadic or familial non-BRCA1/2 patients but tumours of sporadic patients showed more often cyclin B1 expression above 10% than tumours of familial non-BRCA1/2 patients (OR 1.7, 95% CI 1.1–2.7, *P*=0.02).

**Figure 1:**
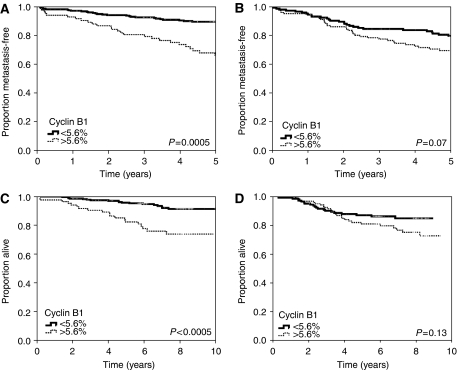
Kaplan–Meier curves showing metastasis-free and overall survival for cyclin B1 dichotomised at 5.6% (7th decile). (**A**) Metastasis-free survival among chemotherapy-naive patients (*n*=473). (**B**) Metastasis-free survival among patients having received adjuvant chemotherapy (*n*=323). (**C**) Overall survival among chemotherapy-naive patients (*n*=473). (**D**) Overall survival among patients having received adjuvant chemotherapy (*n*=473). Kaplan–Meier curves constructed and *P*-values counted comparing the subsets of cases using a log-rank test.

The RRs for OS and MFS were assessed with cyclin B1 dichotomised at 7th decile (5.6%), because we have earlier shown that this is the optimal cut-off for proliferation markers (Ahlin *et al*, 2007). This corresponds to the proportion of grade 3 tumours in our material. The RR for poor survival was 3.74 (95% CI 1.96–7.12, *P*<0.0005) and for metastasis 3.51 (95% CI 2.05–6.01, *P*<0.0005) among chemotherapy-naive patients (Table 3). Among the subgroup of patients that had received chemotherapy, the association with poor MFS was weaker (RR 1.58, 95% CI 0.96–2.60, *P*=0.07) and no significant association with poor OS was found (RR 1.56, 95% CI 0.87–2.80, *P*=0.13). Endocrine treatment did not seem to affect the association of cyclin B1 with survival. RR for poor survival was 3.09 (95% CI 1.68–5.68, *P*<0.0005) among the patients that had received endocrine treatment and 2.24 (95% CI 1.21–4.14, *P*=0.01) among the patients that had not received endocrine treatment. The RRs for metastases were 3.08 (95% CI 1.83–5.18, *P*<0.0005) and 2.06 (95% CI 1.24–3.43, *P*=0.006), respectively. The association with survival was similar when cyclin B1 was analysed as a continuous value (data not shown). In Figure 1, Kaplan–Meier curves show OS and MFS for cyclin B1 dichotomised at 5.6%.

A multivariate model including TNM status, tumour grade, and ER, PR, Ki67, p53 and HER2 status was constructed to analyse the independent impact of cyclin B1 expression on prognosis. High cyclin B1 had an independent association with poor outcome (Table 4). Among chemotherapy-naive patients, the associations were also stronger in multivariate analysis. With tumour size and nodal status, cyclin B1 was the only factor independently associated with poor MFS (RR 2.31, 95% CI 1.17–4.59, *P*=0.016) and OS (RR 1.79, 95% CI 1.28–4.14, *P*=0.04).

## Discussion

This study is so far the most extensive study of cyclin B1 expression in breast cancer and shows that high cyclin B1 expression is a predictor of poor overall and metastasis-free survival. Associations with poor prognosis were stronger among chemotherapy-naive patients. Besides positive nodal status and large tumour size, high cyclin B1 expression was the only independent factor predicting poor OS and MFS among chemotherapy-naive patients. These results suggest that cyclin B1 is a strong independent prognostic marker that could add to accuracy of prognostic evaluation made by traditional prognostic markers and that could easily be adapted for routine use.

Relative risk for poor survival for cyclin B1 was 3.74 and the risk for metastasis 3.51 among chemotherapy-naive patients. In this study, cyclin B1 was a stronger marker of poor prognosis than proliferation markers cyclin A or Ki67 (among chemotherapy-naive patients the RR for poor survival in univariate analysis was 2.47 for cyclin A and 1.90 for Ki67) and the risk for poor survival was also stronger than that has been previously reported for Ki67 or cyclin A. In multivariate analysis among chemotherapy-naive patients, high cyclin B1 was almost as strong predictor of poor OS as HER2 and PR status, or even tumour grade, and it predicted poor MFS more powerfully than HER2 and PR status or tumour grade. The independent prognostic value of cyclin B1 in this study was as strong as or even stronger than the risks reported for commonly used biological markers in breast cancer. The independent relative risk for histological grade has been reported to be approximately 1.70–3.20 (Simpson *et al*, 2000; Elston and Ellis, 2002; Volpi *et al*, 2004), for HER2 2.56 (Joensuu *et al*, 2003), and for the tumour-related proteolytic factors uPA and PAI-1 in a pooled analysis from 18 patient populations 2.58–3.12 (Look *et al*, 2002). In a recent study tumour triple negative status had RR 1.8 for mortality and RR 1.5 for metastasis (Dent *et al*, 2007). Gene expression profiles have been suggested to add specificity to prognostic evaluation made by traditional and immunohistochemical markers. In a validation study, perhaps the most extensively studied profile, the 70-gene prognosis signature predicted metastases with RR of 2.13 (95% CI 1.19–3.82) and mortality with RR of 2.63 (95% CI 1.45–4.79) (Buyse *et al*, 2006). Thus high cyclin B1 might be a biological risk predictor as strong as the 70-gene profile and more easily adapted for routine use.

Our study is the largest so far showing the association of high cyclin B1 and shorter survival in breast cancer. One earlier smaller study with stage I–II tumours did not show any association (Peters *et al*, 2004), one with 109 tumours showed association with only nuclear staining (Suzuki *et al*, 2007) and one with 73 tumours showed a significant association (Winters *et al*, 2001). In the hitherto largest study of 332 tumours, all N-negative, cyclin B1 was associated with poor prognosis but not in multivariate analysis including Ki67 (Rudolph *et al*, 2003), and when 273 tumours with only surgery and postoperative radiation were analysed, cyclin B1 was prognostic only in premenopausal patients (Kühling *et al*, 2003). The limitations of our study include the retrospective setting of the study and the heterogeneity of the patient material concerning adjuvant treatments. Conclusions are, however, in line with the ones suggested by earlier smaller studies, and we feel the role of cyclin B1 as prognostic factor in breast cancer deserves to be further validated, utilising specifically the methods and cut-offs designed in this study.

The correlations between cyclin B1 and other tumour features show that high cyclin B1 expression is common among tumours with an aggressive phenotype. The association of high cyclin B1 expression with large tumour size, positive nodal status, high grade, and ER and PR negativity is similar to previous reported data (Kühling *et al*, 2003; Rudolph *et al*, 2003). One smaller study did not find any correlation to N, T, ER and p53 status, but the small sample size (73 tumours) may explain this discrepancy (Winters *et al*, 2001). In our study, again supported by earlier results, cyclin B1 was strongly associated with high grade and high Ki67, cyclin A and E expression (Winters *et al*, 2001). We also show that high cyclin B1 associated with p53 positivity. This is biologically relevant because p53 controls the cell cycle via cyclin B1 (Innocente *et al*, 1999). Thus multiple biological factors related with an active cell cycle are intercorrelated. Cyclin B1 expression correlated significantly with HER2 positivity and this, to our knowledge, has not been reported earlier and is consistent with an aggressive phenotype. One can speculate on whether the prognostic impact of cyclin B1 reflects only a high tumour proliferation rate or whether high cyclin B1 may reflect also other biological properties of the tumour. In our patients cyclin B1 seems to be a stronger prognostic factor than cyclin A or Ki67. In our material (data not shown), a high cyclin A or Ki67 score was associated with a shorter time to first event among patients eventually developing metastases, whereas the median time to development of metastases was similar in patients with high and low cyclin B1 score. This implies cyclin B1 expression may not be a pure proliferation marker but reflects also other features, for exqample, genomic instability of the tumour as suggested by previous studies (Innocente *et al*, 1999; Shen *et al*, 2004). By analysing both cytoplasmic and nuclear cyclin B1 expression in this study, both aberrant and physiologic cyclin B1 expression were probably included because the cyclin B1/CDK1 complex is relocated to the nucleus only in the beginning of the M phase.

In conclusion, this study shows that cyclin B1 expression is an independent predictor of poor overall and metastasis-free survival in breast cancer. If verified the results of this study suggest cyclin B1 immunohistochemistry is a method that could easily be adapted for routine use as a prognostic marker in breast cancer. The generally lower risk ratios for mortality or metastases in patients given adjuvant chemotherapy suggest that high cyclin B1 score may indicate an enhanced sensitivity to chemotherapy.

## Figures and Tables

**Table 1:** Patient and tumour characteristics

*Grade*	
1	318 (23.6%)
2	606 (45.0%)
3	399 (29.6%)
Not known	25 (1.9%)
	
*Tumour size (T)*	
1	782 (58.0%)
2	448 (33.2%)
3	51 (3.8%)
4	38 (2.8%)
NA	29 (2.2%)
	
*Nodal status (N)*	
Positive	585 (43.4%)
Negative	731 (54.2%)
NA	32 (2.4%)
	
*Primary metastases (N)*	
Positive	48 (3.6%)
Negative	1244 (92.3%)
NA	56 (4.2%)
	
*Clinical stage*	
I	503 (37.3%)
II	659 (48.9%)
III	58 (4.3%)
IV	48 (3.6%)
NA	80 (5.9%)
	
*Oestrogen receptor (ER)*	
Positive	989 (73.4%)
Negative	288 (21.4%)
NA	71 (5.3%)
	
*Progesterone receptor (PR)*	
Positive	828 (61.4%)
Negative	448 (33.2%)
NA	72 (5.3%)
	
*Tumour pathology*	
Ca ductale	924 (68.5%)
Ca lobulare	254 (18.8%)
Ca medullare	20 (1.5%)
Ca mucinosum	25 (1.9%)
Ca papillare	4 (0.3%)
Ca tubulare	46 (3.4%)
Others	75 (5.6%)
	
*HER2*	
Positive	155 (11.5%)
Negative	1074 (79.7%)
NA	119 (8.8%)
	
*p53*	
Positive	253 (18.8%)
Negative	989 (73.4%)
NA	106 (7.9%)
	
*Ki67 expression*	
<5%	318 (23.6%)
5–19%	553 (41.0%)
20–29%	220 (16.3%)
>29%	214 (15.9%)
NA	43 (3.2%)
	
*Age at diagnosis*	
<50 years	493 (36.6%)
⩾50 years	855 (63.4%)
	
*Menopausal status*	
Premenopausal	297 (22.0%)
Postmenopausal	568 (42.1%)
NA	483 (35.8%)

**Table 2:** Correlation of cyclin B1 expression and other tumour characteristics (Spearman's *σ*-correlation test)

	**Correlation coefficient**	**95% CI**	***P* value**
Tumour size (T)	0.163	0.104–0.221	<0.0005
Nodal status (N)	0.081	0.021–0.140	0.008
Primary metastases (M)	0.018	−0.042–0.078	0.56
Clinical stage	0.129	0.075–0.183	<0.0005
Tumour grade	0.493	0.447–0.537	<0.0005
Oestrogen receptor (ER)	−0.327	−0.380– −0.272	<0.0005
Progesterone receptor (PR)	−0.216	−0.273– −0.157	<0.0005
HER2	0.245	0.188–0.301	<0.0005
Triple negativity	−0.275	–0.329– −0.220	<0.0005
Ki67	0.528	0.484–0.570	<0.0005
p53	0.311	0.256–0.364	<0.0005
Age at onset	−0.156	−0.213– −0.098	<0.0005
Menopausal status	−0.164	−0.228– −0.098	<0.0005
Cyclin E	0.392	0.340–0.442	<0.0005
Cyclin D1	0.021	−0.039–0.081	0.50
Cyclin A	0.610	0.571–0.646	<0.0005

Abbreviation: 95% CI=95% confidence interval.

Cyclin B1 analysed as a continuous variable.

^*^P value assessed with the Mann–Whitney *U*-test.

**Table 3:** Cyclin B1 expression and survival in univariate analysis (Cox regression analysis)

	**RR**	**95% CI**	***P* value**
*(A) Metastasis-free survival*			
Chemotherapy naive patients (*n*=473)	3.51	2.05–6.01	<0.0005
All patients (*n*=797)	2.48	1.72–3.57	<0.0005
Chemotherapy patients (*n*=323)	1.58	0.96–2.60	0.07
			
*(B) Overall survival*			
Chemotherapy naive patients (*n*=473)	3.74	1.96–7.12	<0.0005
All patients (*n*=797)	2.58	1.82–3.90	<0.0005
Chemotherapy patients (*n*=323)	1.56	0.87–2.80	0.13

Abbreviations: RR=relative risk; 95% CI=95% confidence interval.

Cyclin B1 dichotomised at 7th percentile (5.6%).

**Table 4:** Cyclin B1 expression and survival in multivariable analysis (Cox regression analysis)

	**RR**	**95% CI**	***P* value**
*(A) Overall survival*			
* Chemotherapy naive patients (*n*=473)*			
Nodal status	3.41	1.55–7.49	0.002
Tumour size	2.87	1.95–4.22	<0.0005
Cyclin B1	1.80	1.28–4.14	0.04
HER2	2.03	0.82–5.02	0.126
Ki67	1.59	0.82–3.79	0.20
Progesterone receptor	1.75	0.73–4.17	0.21
Oestrogen receptor	1.70	0.52–5.55	0.38
Grade	1.43	0.78–2.63	0.25
p53	1.15	0.74–1.80	0.54
			
* All patients (*n*=797)*			
Nodal status	4.17	2.33–7.49	<0.0005
Progesterone receptor	1.99	1.10–3.61	0.02
HER2	1.91	1.15–3.18	0.01
Tumour size	1.87	1.47–2.37	<0.0005
Grade	1.79	1.14–2.79	0.01
Cyclin B1	1.83	0.99–3.40	0.05
p53	1.15	0.91–1.47	0.25
Ki67	1.08	0.62–1.25	0.28
Oestrogen receptor	0.89	0.45–1.76	0.74
			
*(B) Metastasis-free survival*			
*Chemotherapy naive patients (*n*=473)*			
Nodal status	2.76	1.48–5.13	0.001
Cyclin B1	2.31	1.17–4.59	0.02
Tumour size	1.91	1.35–2.72	<0.0005
Grade	1.44	0.90–2.32	0.13
Ki67	1.39	0.82–1.65	0.35
Progesterone receptor	1.27	0.60–2.67	0.53
Oestrogen receptor	1.10	0.36–3.36	0.87
HER2	1.10	0.46–2.63	0.82
p53	1.06	0.72–1.55	0.79
			
* All patients (*n*=797)*			
Nodal status	2.97	1.87–4.67	<0.0005
Cyclin B1	1.68	1.02–2.74	0.04
Tumour size	1.64	1.33–2.03	<0.0005
Grade	1.63	1.14–2.32	0.008
HER2	1.46	0.92–2.31	0.11
Progesterone receptor	1.39	0.84–2.31	0.20
Ki67	1.26	0.67–1.52	0.26
p53	1.14	0.92–1.41	0.23
Oestrogen receptor	0.81	0.45–1.47	0.49

Abbreviations: RR=relative risk; 95% CI=95% confidence interval.

Cyclin B1 dichotomised at 7th percentile (5.6%).
